# Time-dependent inhibition of Rac1 in the VTA enhances long-term aversive memory: implications in active forgetting mechanisms

**DOI:** 10.1038/s41598-023-40434-9

**Published:** 2023-08-19

**Authors:** Juliana F. Dalto, Jorge H. Medina

**Affiliations:** 1 Instituto de Biología Celular y Neurociencias “Prof. Eduardo de Robertis”, Facultad de Medicina, Universidad de Buenos Aires-CONICET, Paraguay 2155, 3rd Floor, C1121ABG Buenos Aires, Argentina; 2 Instituto Tecnológico de Buenos Aires, Buenos Aires, Argentina

**Keywords:** Neuroscience, Learning and memory, Forgetting

## Abstract

The fate of memories depends mainly on two opposing forces: the mechanisms required for the storage and maintenance of memory and the mechanisms underlying forgetting, being the latter much less understood. Here, we show the effect of inhibiting the small Rho GTPase Rac1 on the fate of inhibitory avoidance memory in male rats. The immediate post-training micro-infusion of the specific Rac1 inhibitor NSC23766 (150 ng/0.5 µl/ side) into the ventral tegmental area (VTA) enhanced long-term memory at 1, 7, and 14 days after a single training. Additionally, an opposed effect occurred when the inhibitor was infused at 12 h after training while no effect was observed immediately after testing animals at 1 day. Control experiments ruled out the possibility that post-training memory enhancement was due to facilitation of memory formation since no effect was found when animals were tested at 1 h after acquisition and no memory enhancement was observed after the formation of a weak memory. Immediate post-training micro-infusion of Rac1 inhibitor into the dorsal hippocampus, or the amygdala did not affect memory. Our findings support the idea of a Rac1-dependent time-specific active forgetting mechanism in the VTA controlling the strength of a long-term aversive memory.

## Introduction

More than a century ago, James^1^ asked whether we can explain differences in the duration of memories. Why do some of them last minutes while others can be recalled for a few days, and others can last weeks, months and even a lifetime? To answer this question, we must answer first how, when, and where stored information persists and/or decays in a time-dependent manner. This leads to studying active processes of memory persistence and forgetting^2–5^. In fact, and according to several authors^4,6,7^, there is a continuous balance between opposing active processes that determines if long-term memory (LTM) will be maintained or forgotten. Forgetting could be defined as the inability to access a memory which was previously acquired and retrieved^7^. Although forgetting is usually considered as an impairment, it is in fact a fundamental process for selecting those memories that will remain and occasionally will drive advantageous behavior. Traditional theories suggest that forgetting occurs because of retroactive interference from other cognitive functions or due to natural decay of the memory trace^8,9^. In the last few years attention has been focused on the molecular mechanisms of active decay of memory storage. More specifically, some molecules and intracellular pathways have been postulated to participate in what is referred to as active forgetting (see for references, Davis and Zhong^4^ Frankland et al.^6^ Medina^7^). One of these molecules is Rac1. This small protein, a member of the Rho family of GTPases, is involved in synaptic remodeling due to its role in cytoskeletal organization^10^. Rac1 is activated by Ca^2+^ via the activation of NMDA receptors/CaMKII and/or BDNF/TrkB signaling pathways and sequentially activates PAK and LIMK which in turn inactivate cofilin^9,11–13^. Rac1 signaling pathway is involved in the polymerization, elongation and stabilization of the actin cytoskeleton within activated dendritic spines, thereby modulating memory formation and active forgetting^9,14,15^. Several studies in *Drosophila* and mice have demonstrated that Rac1 activity is important as an active mechanism of forgetting in some forms of memory^14,16,17^. In *Drosophila*, Rac1 and ERK1/2 pathways play a role in the forgetting of a labile and transient aversive memory lasting some hours^14,18^. However, in rodents, the evidence concerning the role of Rac1 in the forgetting of consolidated aversive memories is not clear. While Rac1 activity in the hippocampus (HP) appears not to participate in the active forgetting of aversive memories in mice^17^, a recent paper showed that hippocampal Rac1 activity controls forgetting of a contextual fear conditioning^19^. Based on these considerations, the main objective of the present study is to determine whether Rac1 activity in selected brain regions is required for modulating long-lasting memory storage of a single-trial inhibitory avoidance (IA) learning, a well-studied aversive task in rats^20–22^.

## Results

### Inhibition of Rac1 specifically in the VTA enhances IA LTM

**Figure 1:**
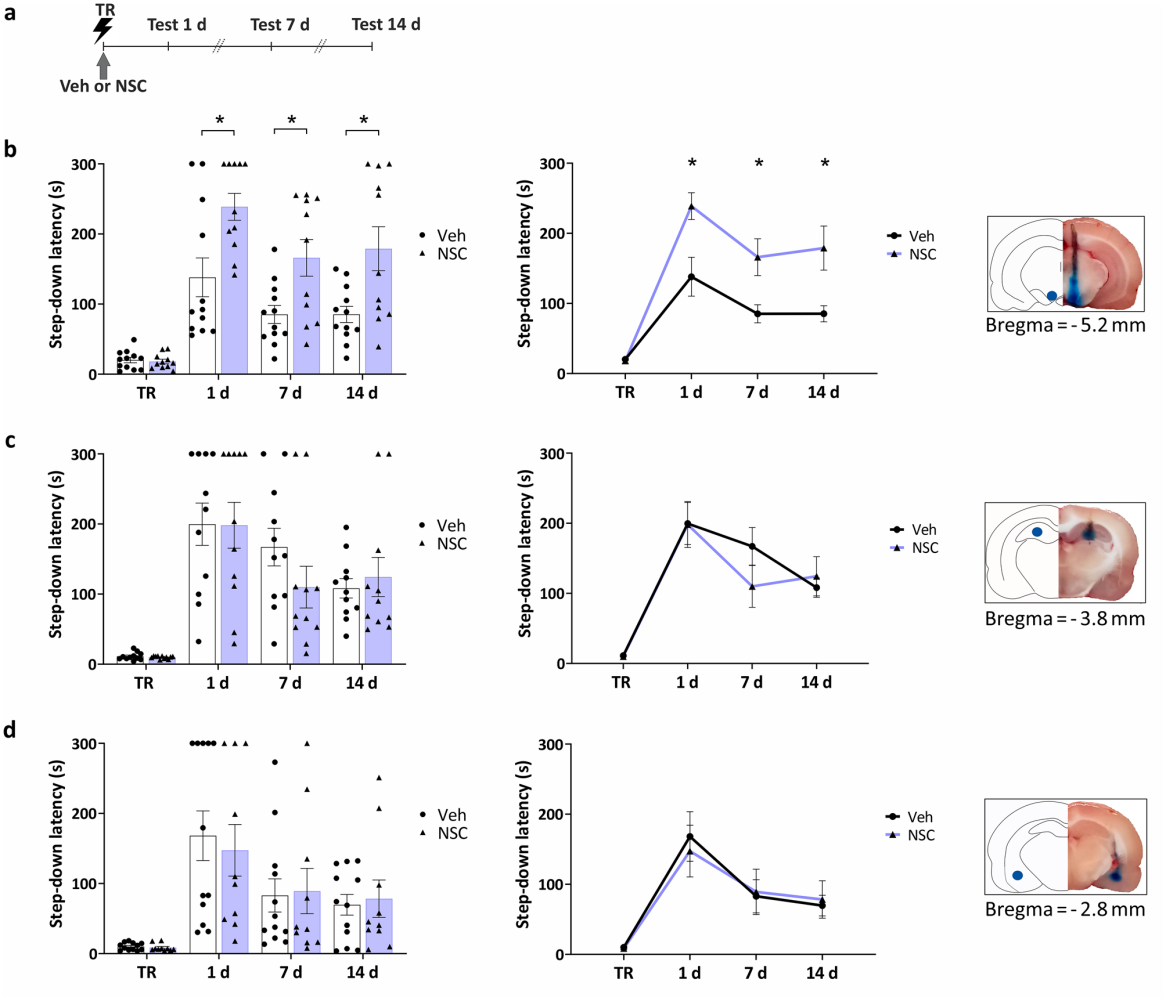
Post-training inhibition of Rac1 in the VTA, but not in the HP or the AMG, enhanced IA LTM at 1, 7 and 14 days after training. (**a**) Experimental schedule. Animals were trained (TR) and tested by step-down latency measurement at 1, 7 and 14 days. Vehicle (Veh) or the Rac1 inhibitor NSC23766 was infused immediately after training (arrow). (**b**)–(**d**) Individual data points (left panel) and line (middle panel) graphs. Infusion of NSC23766 into the VTA significantly enhanced memory at every time (b; *n*_Veh_ = 12, *n*_NSC_ = 11) while no effect was observed when the infusion was made into the HP (c; *n*_Veh_ = *n*_NSC_ = 11) or the AMG (d; *n*_Veh_ = 12, *n*_NSC_ = 10). Right panel. Representative schemes (left) and photographs (right) of a coronal section of the rat brain showing the aimed cannula placement and methylene blue diffusion. Data are presented as means ± SEM. *p  < 0.05 from Mann–Whitney U for each test.

Based on previous findings from our laboratory showing that the VTA^23^ and the HP^24^ promote active forgetting of a rewarding single-trial cocaine-place conditioning in rats, and that hippocampal Rac1 seems to play a role in active forgetting in mice^17^, we first studied the effect of the inhibition of Rac1 activity in 3 selected brain regions known to be important for IA memory processing: the VTA, the HP and the amygdala (AMG)^22,25^. As shown in Fig. 1, the immediate post-training infusion of NSC23766 (150 ng/0.5 ul/side) into the VTA (Fig. 1b , 1 day: *U* = 26, *p* = 0.0118; *n*_Veh_ = 12, *n*_NSC_ = 11), but not into the dorsal HP (Fig. 1c, 1 day: *U* = 60, *p* > 0.9999; 7 days: *U* = 36, *p* = 0.1108; 14 days: *U* = 54, *p* = 0.6853; *n*_Veh_ = *n*_NSC_ = 11) or the AMG (Fig. 1d, 1 day: *U* = 56,60, *p* > 0.8282; 7 days: *U* = 57, *p* = 0.8718; 14 days: *U* = 59, *p* = 0.9742; *n*_Veh_ = 12, *n*_NSC_ = 10), enhanced LTM 1 day after training. Furthermore, memory enhancement is maintained at 7 and 14 days tests (Fig. 1b, 7 day: *U* = 29, *p* = 0.0225; 14 days: *U* = 31, *p* = 0.0310).

### Does Rac1 induce an active forgetting mechanism, or down-regulate memory formation?

**Figure 2:**
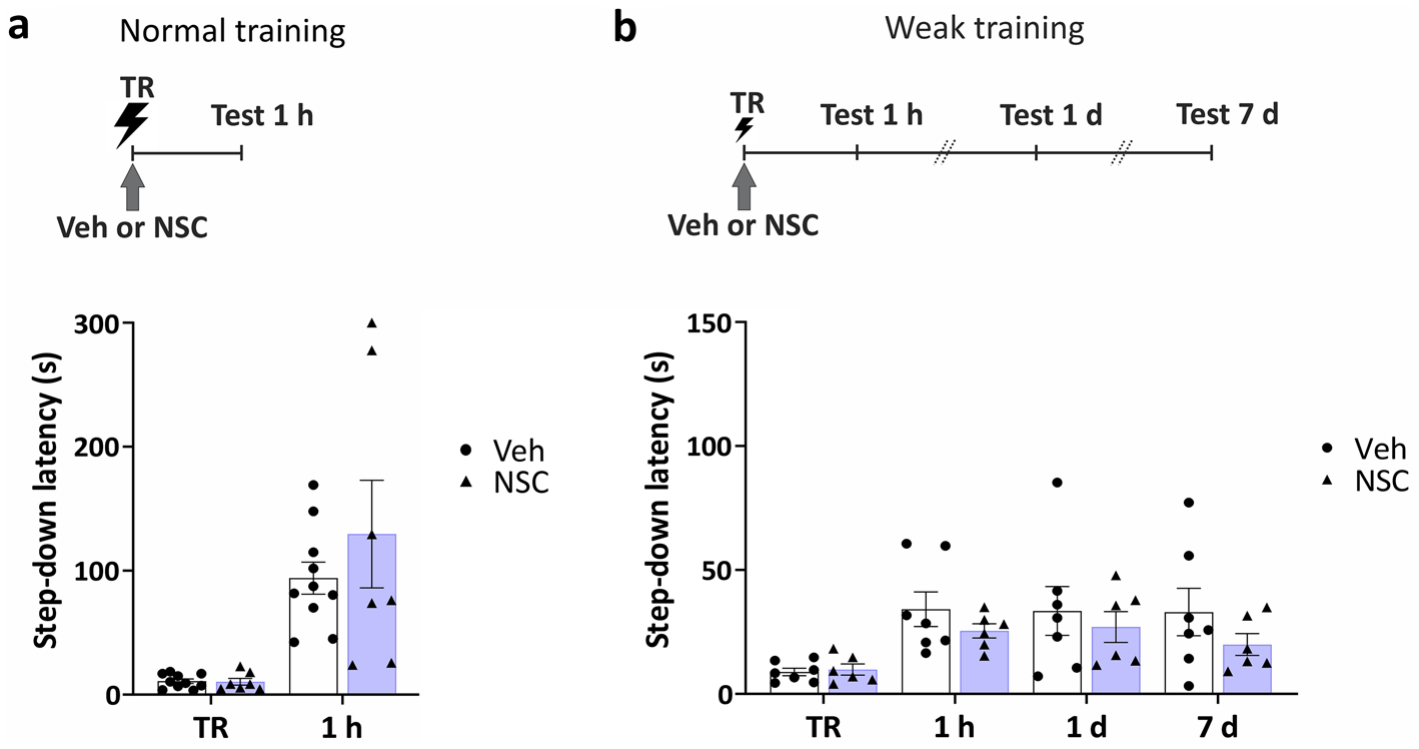
Post-training inhibition of Rac1 in the VTA had no effect on IA memory formation. (**a**) The infusion of Rac1 inhibitor NSC23766 into the VTA immediately after a normal training (0.36 mA), did not affect memory expression at 1 h. *n*_Veh_ = 10, *n*_NSC_ = 7. (**b**) The infusion of Rac1 inhibitor NSC23766 into the VTA immediately after a weak training (0.26 mA), did not affect memory expression at 1 h nor memory maintenance at 1 and 7 days. Note that the scale in the graph is smaller. *n*_Veh_ = 7, *n*_NSC_ = 6. Data are presented as means ± SEM.

To determine whether the enhanced memory expression observed with Rac1 inhibition was due to protecting IA memory from forgetting rather than facilitating memory formation, we conducted two control experiments. First, we tested recent memory after the immediate post-training inhibition of Rac1 activity in the VTA. We confirmed that all animals expressed IA memory at 1 h (Fig. 2a, Veh: *W* = 55, *p* = 0.0010, *n*_Veh_ = 10; NSC: *W* = 28, *p* = 0.0078, *n*_NSC_ = 7) which was not modified by NSC23766 infusion (Fig. 2a, 1 h: *U* = 34, *p* = 0.9623). This is consistent with the notion that Rac1 activity has no impact on memory formation in rodents.^17,26,27^ Second, with the aim of sensitizing the experimental protocol to allow a better visualization of a potential facilitatory effect on memory formation, we used a weak foot-shock in the training session*.* The prediction of using a weak IA protocol is that a facilitatory effect of a given compound on memory formation should be equal to or greater than that observed when using a stronger IA protocol.^25,28,29^ As expected, rats subjected to a weak IA protocol showed poor memory (Fig. 2b, Veh: 1 h vs TR: *W* = 28, *p* = 0.0078, 1 day vs TR: *W* = 28, *p* = 0.0078, *n*_Veh_ = 7; NSC: 1 h vs TR: *W* = 21, *p* = 0.0156, 1 day vs TR: *W* = 19, *p* = 0.0313, *n*_NSC_ = 6). However*,* in contrast to the above mentioned prediction, the immediate post-training infusion of NSC23766 into the VTA in rats subjected to a weak IA protocol did not induce any enhancement of IA memory (Fig. 2b, 1 h: *U* = 16, *p* = 0.5338; 1 day: *U* = 21, *p* > 0.9999; 7 days: *U* = 15, *p* = 0.4452).

### Memory enhancement induced by Rac1 inhibition in the VTA is time-dependent

**Figure 3:**
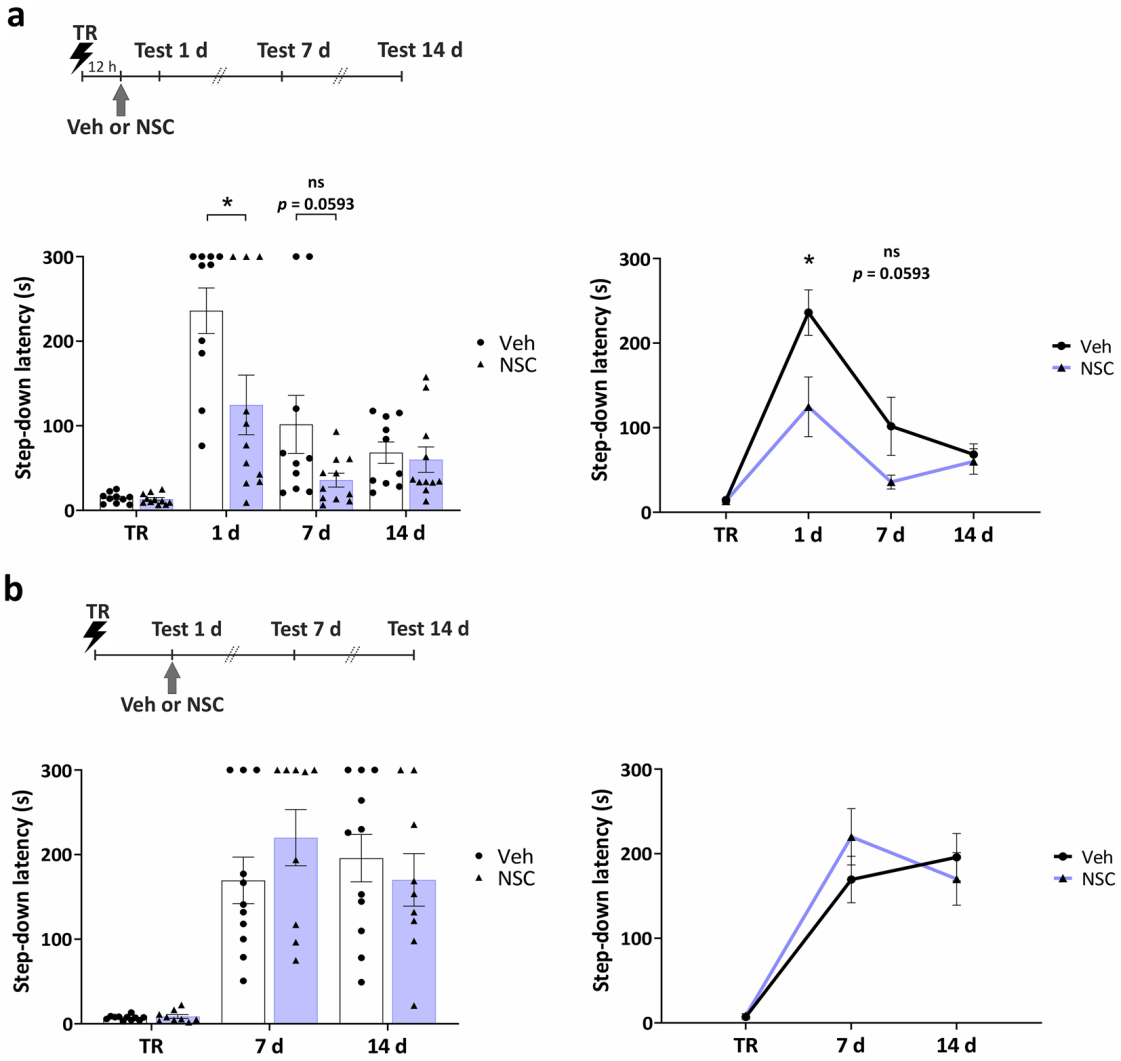
Inhibition of Rac1 in the VTA has different effects on IA LTM depending on the time after training. (**a**) Individual data points (left) and line (right) graphs. Infusion of Rac1 inhibitor NSC23766 into the VTA at 12 h after training significantly decreased memory expression at 1 d. *n*_Veh_ = 10, *n*_NSC_ = 11. (**b**) Individual data points (left) and line (right) graphs. Infusion of Rac1 inhibitor NSC23766 into the VTA immediately after 1-d test, had no effect on memory expression. *n*_Veh_ = 11, *n*_NSC_ = 9. Data are presented as means ± SEM. *p < 0.05 from Mann–Whitney U for each test.

To assess whether there were other periods at which the infusion of NSC23766 into the VTA could induce memory enhancement, we infused the Rac1 inhibitor at several time points after acquisition. We first assessed the effect of the infusion of NSC23766 at 12 h after training since previous studies have shown the relevance of this time point for the persistence but not formation of aversive memories^21,22^. Rac1 inhibition at this specific time did not provoke memory enhancement but instead decreased memory expression at 1 d (Fig. 3a, 1 day: *U* = 27, *p* = 0.0471; 7 days: *U* = 28, *p* = 0.0593; 14 days: *U* = 52, *p* = 0.8633; *n*_Veh_ = 10, *n*_NSC_ = 11) ruling out the possibility that memory enhancement induced by the immediate administration of NSC23766 into the VTA (see Fig. 1b) was caused by a protracted action on memory retrieval. Also, we wondered whether a memory enhancement could be observed when Rac1 activity was inhibited immediately after retrieval since a previous work showed that manipulation of Rac1 activity in the HP at this time point improved memory expression for a contextual fear conditioning in mice^19^. We first confirmed memory expression and homogeneity between groups at 1 d (Veh: *W* = 66, *p* = 0.0005; NSC: *W* = 45, *p* = 0.0020; 1 day: *U* = 34, *p* = 0.1716, *n*_Veh_ = 11, *n*_NSC_ = 9). Then we assessed the effect of the infusion of NSC23766 into the VTA immediately post retrieval and found no effect on IA memory expression at 7 and 14 days. (Fig. 3b, 7 days: *U* = 39, *p* = 0.4453; 14 days: *U* = 44, *p* = 0.7077).

### Rac1 inhibition in the VTA has no effect on locomotor activity or anxiety-like behavior

**Figure 4:**
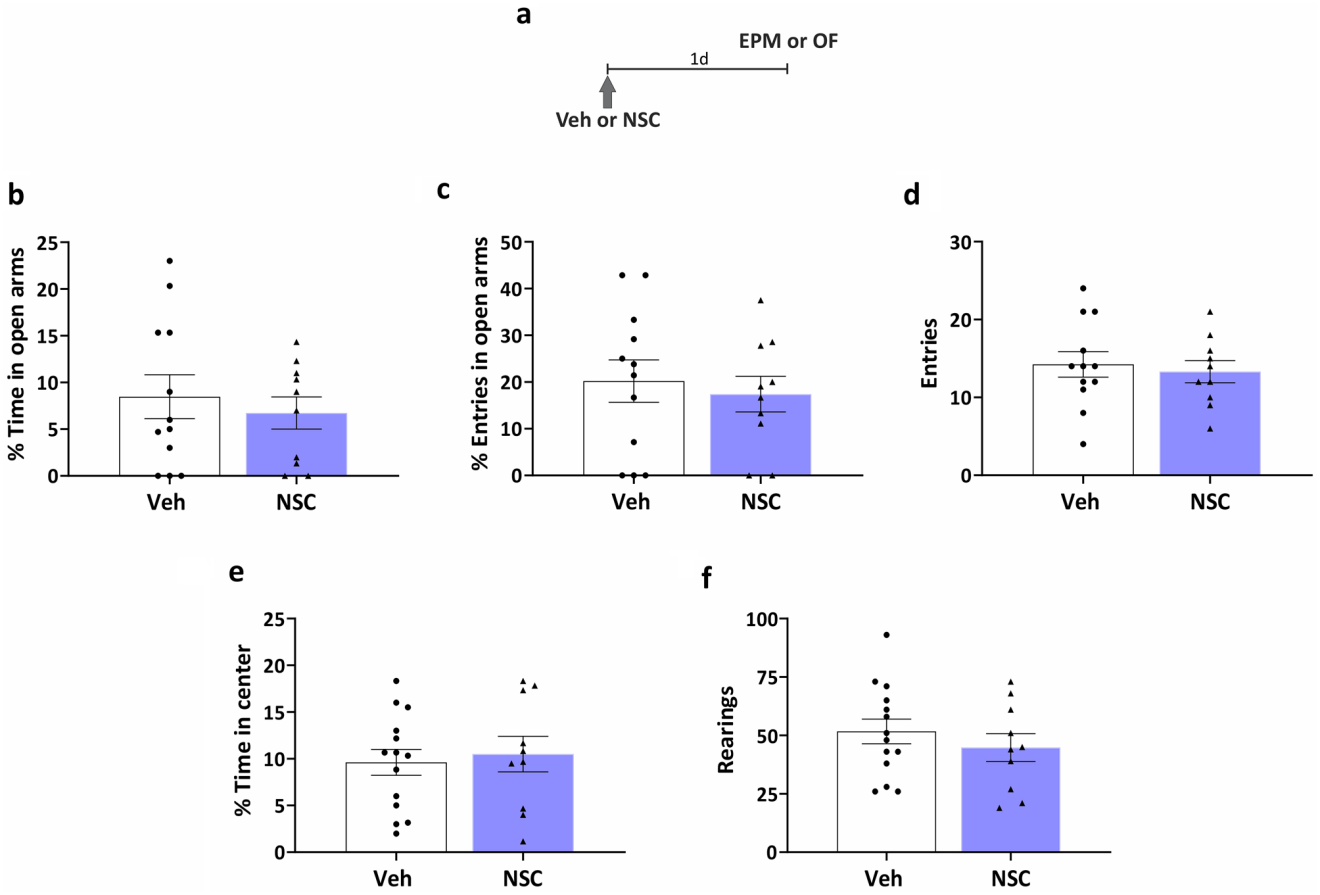
Inhibition of Rac1 in the VTA does not affect exploratory or anxiety-like behavior. (**a**) Experimental schedule. Animals were infused (arrow) with vehicle (Veh) or the Rac1 inhibitor NSC23766 and tested 1 d later in the elevated plus maze (EPM, b-d) or the open field (OF, e–f) tasks. (**b**) Percentage of time spent in the open arms. (**c**) Percentage of entries in the open arms. (**d**) Total number of entries to any of the arms. (**e**) Percentage of time spent in the center of the open field. (**f**) Total number of rearings in the open field. *n*_Veh_ = 12–14, *n*_NSC_ = 10. Data are presented as means ± SEM.

Given that anxiety-like behavior in mice is controlled by VTA projections to AMG^30^ and lateral septum^31^, we next determined whether the inhibition of Rac1 in the VTA could induce anxiety-like behavior 1 d later (Fig. 4a). Rats infused with NSC23766 displayed similar performance to control animals in the EPM (Fig. 4b–d, (b) t_(20)_ = 0,58, *p* = 0.5691. (c) t_(20)_ = 0.46, *p* = 0.6515. (d) t_(20)_ = 0.43, *p* = 0.6718, *n*_*veh*_ = 12, *n*_*NSC*_ = 10) as well as in the OF task (Fig. 4e, f, (e) t_(22)_ = 0.38, *p* = 0.7054. (f) t_(22)_ = 0.86 *p* = 0.3989, *n*_*veh*_ = 14, *n*_*NSC*_ = 10). These results rule out the possibility that higher latency observed from 1 d after IA training in animals infused with NSC23766 (see Fig. 1a) was driven by differences in anxiety or locomotor activity.

### ERK 1/2 signaling is not involved in memory enhancement induced by Rac1 inhibition

**Figure 5:**
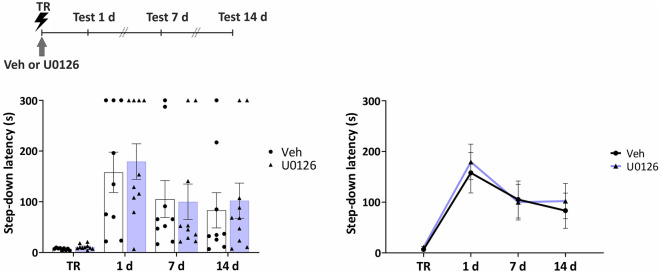
Post-training inhibition of MEK1/2 in the VTA did not affect IA LTM. Individual data points (left) and line (right) graphs. Infusion of MEK 1/2 inhibitor U0126 into the VTA immediately after training had no effect on memory expression. *n*_Veh_ = 9, *n*_NSC_ = 10. Data are presented as means ± SEM.

Rac1 activates an important number of signaling pathways, including among others, those that modulate signal transduction and protein synthesis regulated by MEK1/2/ERK1/2 pathway^32–34^. In addition, ERK 1/2 modulates forgetting of a transient aversive memory in *Drosophila*^9,18^. Therefore, we infused the selective MEK1/2 inhibitor U0126 (0.25 μg/0.5 μl/side) into the VTA, a dose that consistently affects memory formation and persistence in rats^23,35^. No effect on memory retention scores was found at any time point tested (Fig. 5, 1 day: *U* = 39, *p* = 0.6513; 7 days: *U* = 42, *p* = 0.8416; 14 days: *U* = 37, *p* = 0.5481; *n*_Veh_ = 9, *n*_NSC_ = 10).

## Discussion

### Memory enhancement by inhibition of Rac1 in the VTA

The main finding of the present study is that Rac1 activity in the VTA, but not in the HP and the AMG, could be part of an active mechanism that attenuates consolidated memory of an aversively-motivated learning task in rats, as it has been demonstrated in several memory tasks in *Drosophila* and mice^4^. Inhibition of Rac1 in the VTA immediately after training induced a time-dependent enhancement of memory performance lasting at least 14 days (Fig. 1b). This enhancement does not seem to be attributable to a facilitating effect on memory consolidation process since no facilitatory effect on memory formation was seen 1 h after training (Fig. 2a), or at any time point tested when a weak training protocol is used (Fig. 2b). This last finding suggests that inducing a weak IA LTM appears not to require a Rac1-dependent mechanism of memory decay*.* Alternatively, our present findings cannot totally rule out the possibility that facilitation of IA memory formation participates, at least in part, in the enhancing effect of IA memory induced by VTA Rac1 inhibition. Future experiments using tests at longer time intervals (1–3 months after training) might help elucidate the mechanisms involved. Our present findings together with the results demonstrating that dopamine neurotransmission in the VTA participates in the active forgetting of a rewarding task^23^, suggest that the VTA may be an important brain component of the intrinsic mechanisms of forgetting in rats. To confirm the existence of an active forgetting mechanism of a consolidated memory two predictions must be met: 1-inhibition, abolition, or attenuation of an active mechanism of forgetting should result in an enhancement in memory expression or in the persistence of memory storage; 2- stimulation or facilitation of the very same mechanism of forgetting should result in memory loss or attenuation well below the normal strength and decay of that memory. Both predictions need additional requirements to consider them fulfilled. Facilitation of memory formation (in prediction #1) or inhibition of memory formation (in prediction #2) must be ruled out respectively to further demonstrate the existence of an inherent forgetting process. Only when information endorsing both predictions and requirements is available, one can be sure that a mechanism of time-dependent active forgetting is present^4,7^. To the best of our knowledge these predictions and requirements were totally achieved by only a few molecular mechanisms in *Drosophila* and in rodents^4,7^. In this regard, to fully confirm that Rac1 activity in the VTA is a key player in forgetting consolidated IA memory, the study of the role of Rac1 activators on IA training will be required. It is important to stress here that available Rac1 activators facilitate not only Rac1 but also Cdc42 and RhoA activities. Thus, due to the lack of specificity of the current commercial reagents it is less feasible to accurately assess the effect of Rac1 activators.

### Rac1 and active forgetting

The first direct evidence for the existence of an active intrinsic mechanism of forgetting was provided by Zhong and colleagues^14^. By using olfactory aversive conditioning in *Drosophila* these authors found that Rac1 is an important molecular target in forgetting a labile aversive component of olfactory memory. Inhibition of Rac1 induced a decrease in early memory decay, increasing its duration from a few hours to more than one day. Conversely, activation of Rac1 promoted forgetting of that labile aversive component of olfactory memory. Some findings in rodents support the idea that mechanisms of active forgetting involve the activation of small Rho GTPases. It has been shown that inhibition of hippocampal Rac1 in mice maintains object recognition memory for 5 days, a memory that normally lasts no more than 1–2 days, whereas activation of Rac1 facilitates forgetting^17^. These authors also reported that modulation of Rac1 activity in the HP did not alter contextual fear conditioning. In marked contrast, administration of NSC23766 improves acquisition and memory formation of contextual fear conditioning in rats^36^. Unfortunately, in this study there has been no control experiment to demonstrate that the increase in the expression of contextual fear memory is due to the facilitation of memory consolidation or to the inhibition of an active forgetting mechanism. Also, discrepancies between those works may be due to differences in training protocols, the type, and dynamics of alterations in hippocampal Rac1 activity and in the timing of Rac1 activity changes with respect to the different stages of memory processing. Our results showed that Rac1 inhibition in the HP did not affect IA memory retention scores (Fig. 1c). This is consistent with those demonstrating that inhibition of Rac1 in the dorsal HP did not alter memory performance of other aversive tasks such as contextual fear conditioning^17^, but are at odds with an elegant report showing that the inhibition of Rac1 in the HP of mice enhances memory expression at 1 and 7 days after a single-trial contextual fear conditioning^19^. Methodological issues, such as the use of different species and learning paradigms could help explain the discrepancies between our results and those reported by Zhong’s group^19^. Given the works so far published on Rac1 and forgetting, it is worthy to mention here that while Rac1 activity plays a role in a labile aversive component of an olfactory conditioning in *Drosophila*, this small GTPase mainly regulates forgetting of consolidated memories in rodents.

### VTA in memory processing and forgetting: the role of dopamine neurotransmission

Since the pioneering work of Lisman and Grace^38^ regarding the role of VTA-hippocampal loop in controlling the information into LTM, much attention has been focused on the participation of the VTA in memory processing. It has been shown that the activation of VTA and its subpopulations of dopaminergic, glutamatergic, and GABAergic neurons modify behaviors and participate in LTM formation, persistence and forgetting of various types of learning paradigms in rodents. Briefly, we can summarize the mnemonic role of this important brain region in: 1- Dopamine neurotransmission in the VTA as well as in the HP facilitates memory formation^39–41^. 2- Dopamine in the VTA and in the HP acting on D1 receptors facilitates persistence of IA memory^22,41^, but importantly does not induce forgetting^41^. 3- In contrast, Dopamine acting on Gq/PLC-linked DA receptors (probably D5 receptors) in the rat HP is a key component of the mechanism of forgetting of a cocaine-associated memory^24^. Moreover, Dopamine neurotransmission in the VTA controls forgetting of the same reward memory^23^. 4- GABAergic and dopaminergic neurons in the VTA drive conditioned place aversion^41,42^. 5- Importantly, acquisition and processing of aversive stimuli activates VTA in humans^43^. Our present findings together with the above-mentioned results support the notion that the VTA is an important brain component of memory processing in rats. Also we highlight the time- and region-specificity of the Rac1-dependent mechanism triggered in the VTA and impacting on an aversive learning task.

### Molecular pathways involved in Rac1 signaling

Regarding the molecular pathways involved in Rac1 regulation, in *Drosophila*, a dopamine/DAMB dopamine receptor/scribble/Rac1 signaling pathway was proposed^16,44^. In rodents, there is no experimental data regarding which the upstream modulators of Rac1 are to mediate forgetting. Moreover, we recently showed that the inhibition of D1/D5 dopamine receptors in the VTA and the HP results in an impairment of the formation or persistence of consolidated aversive memories such as IA and conditioned place aversion memories in rats^41^. This evidence together with our present findings suggests separate roles of dopamine and Rac1 in the VTA for the maintenance of IA memory storage. However, we did not explore the possible role of dopamine in the AMG in similar experiments. Thus, it would be interesting to analyze in the near future whether the modulation of dopamine neurotransmission in this brain region could regulate the IA memory enhancement induced by Rac1 inhibition in the VTA. What are the downstream effectors involved in the forgetting effect of Rac1? In *Drosophila*, Rac1 recruits both actin polymerization and depolymerization pathways including PAK/LIMK/cofilin to downregulate memory storage^14,16,45^. In contrast, in rodents no evidence regarding the downstream effectors of Rac1 to mediate forgetting has been provided, so far. In addition to PAK signaling pathway, in vitro studies have evidenced a width variety of Rac1 effectors including Dia and WAVE-activated Arp 2/3 that initiate the assembly of filamentous actin^46^, lipid kinases, PLC and ROCK1 to regulate endocytosis^47^, p38 MAPK, MEK ½^34^, PKA, PKC, receptor tyrosine kinases, NADPH oxidase, transcription factor STAT3, and beta-catenin^48–50^. Therefore, to determine which of the Rac1 effectors is involved in forgetting aversive learning tasks further experiments are needed.

### Rac1 involvement in other memory processes

Rac1 activity plays a role not only in active forgetting but also in acquisition and memory formation of several learning tasks. Rac1/PAK pathway promotes cofilin phosphorylation and prevents depolymerization of actin filaments. Interfering with actin polymerization did not alter short-term memory but impeded the consolidation of long-term fear memory^26,51^. Conversely, Rac1 activation is related with rapid encoding of associative fear learning^11^ while pharmacological inhibition of Rac1 impairs LTP and LTD in CA1 neurons of the HP and the loss of Rac1 induces a deficit in the acquisition of spatial memory without affecting long-term memory^52^. In the AMG, selective inhibition of Rac1 abolished short-term and long-term memory for fear conditioning^26,53^. Furthermore, knocking down Rac1 expression in rat prefrontal cortex abolished conditioning place aversion extinction, whereas activation of Rac1 accelerated place aversion extinction^37^. In this context, the deleterious effect on memory expression caused by the inhibition of Rac1 in the VTA at 12 h after training (Fig. 3a) could be related to a late period after acquisition involved in memory processing of different learning tasks including IA^21,22^. Our finding showed that Rac1 activity in the VTA participates late after training to partially sustain LTM and adds to a series of molecular mechanisms that maintain LTM storage (see Medina^7^ for references). Therefore, it appears that Rac1 and related regulatory proteins of actin cytoskeleton is crucially involved in both long-term memory formation^12,15^ and forgetting^14,16,17,27,54^. To explain some discrepancies in the literature about actin dynamics and forgetting, critical factors to be further analyzed in the near future include the type of experience to be acquired, at which stage of memory processing occurs the experimental intervention on signaling pathways and which molecular target is chosen to study the role of actin dynamics on memory processing. Also, those above-mentioned findings highlight the need for a clear experimental distinction between the inhibition of the mechanisms of forgetting and the facilitation of the mechanisms of memory formation. In conclusion, a great body of evidence supports the idea that controlled Rac1 activity in invertebrates and mammals regulates the strength of different types of memory and that manipulating Rac1 activity affects the maintenance of memory storage and can lead to transient or permanent forgetting.

## Methods

### Animals

Experiments were performed in adult male Wistar rats of two months old weighing 200–250 g on arrival at the laboratory. Animals were housed in groups of three with water and food ad libitum and maintained on a 12 h direct light–dark cycle (lights on at 7:00 h) at a constant temperature of 21 °C. Behavioral procedures took place during the light phase of the cycle. Experimental procedures followed the Animal Research: Reporting of In Vivo Experiments (ARRIVE) guidelines and were approved by the Animal Care and Use Committee of the University of Buenos Aires (CICUAL). All methods were performed in accordance with the relevant guidelines and regulations.

### Surgical procedure

Each rat was anesthetized with a mix of ketamine (80 mg/kg) and xylazine (8 mg/kg) administered intraperitoneally and placed in a stereotaxic frame. The skull was exposed and aligned (flat skull, lambda and bregma at the same elevation degree) and 22-G guide cannulae (measuring 1 cm length) for intracerebral infusions were bilaterally implanted aiming at different structures. The stereotaxic coordinates used were as follows: for ventral tegmental area (VTA): DV-7.20 mm/AP-5.30 mm/L ± 1.00 mm; for dorsal hippocampus (HP): DV-3.00 mm/AP-3.90 mm/L ± 3.00 mm; for amygdala (AMG): DV-6.80 mm/AP-2.80 mm/L ± 4.80 mm from bregma^55^. Cannulae were fixed to the skull with acrylic cement. Immediately after surgery, animals were injected with a single subcutaneous dose of meloxicam (0.2 mg/kg) and gentamicin (3 mg/kg) as analgesic and antibiotic respectively and were left to recover in their home cage for 1 week.

### Drugs

NSC23766 (Tocris Bioscience, Bristol, UK) and U0126 (Sigma-Aldrich, St. Louis, MO, USA) were dissolved in DMSO 5% in saline and infused bilaterally at a concentration of 150 ng/0.5 μl/side and 0.25 μg/0.5 μl/side respectively. The doses used in the present study were based on in vitro IC50 and on previous in vivo works^56,57^. NSC23766 is a small molecule that acts as a Rac1-selective inhibitor in non-neuronal cells^58^ as well as in the dorsal HP and other brain regions^59^. It represents the first generation of Rac1-specific inhibitors, highly soluble and membrane permeable^48^. U0126 was used as an ERKs1/2 inhibitor given that it blocks the kinase activity of MEK1/2, thus preventing the activation of ERKs1/2.

### Drug intracerebral infusion

For intracerebral infusions, 30-G needles connected to Hamilton syringes were used (1.2 cm length for the VTA and the AMG, 1.1 cm length for the HP). The infusions were always bilateral (0.5 μl/side; infusion rate: 1 μl/20 s). The entire infusion procedure took around 2 min including the infusions themselves and the handling. The needle was left in place for 45 s after infusion was completed to allow diffusion and to prevent reflux. At the end of each experiment, the placement of the drug infusion was verified by infusions of 0.5 μl of methylene blue 4% in saline. Animals were killed by CO_2_ inhalation and the brain was removed for histological localization of the infusion site. The extension of the dye infused was taken as indicative of the presumed diffusion of the drugs previously given to each animal. Only animals with both cannulae in the correct place were included in the study.

### Behavioral paradigm—inhibitory avoidance (IA) task

During the training session, each rat was placed on a 5 cm high, 9 cm wide platform placed on the left of a 47 × 25 × 30 cm3 opaque acrylic box, with a grid floor. As they stepped down onto the grid with all four paws, they received a scrambled foot shock for 3 s (0.36 or 0.26 mA). Immediately after, animals were removed from the apparatus. Rats were infused with vehicle or the Rac1 inhibitor NSC23766 into the VTA, the HP or the AMG at different time points after training depending on the experiment (immediately after training, 12 h after training, immediately after 1-d test session). Except for the experiments where rats were tested at 1 h (see Fig. 2), animals were tested for memory retention three times after training at 1, 7 and 14 days. The procedure was similar to the training except no foot-shock was given. Latency to step down from the platform was measured in both training and test sessions. A limit of 300 s was imposed on retention test measures, so the test sessions were finished either when the rat stepped down from the platform or when the limit was reached. In either case, the rat was immediately removed from the apparatus. No signs of memory extinction were seen in our experiments using long-spaced test sessions (once every 6–7 days), which is consistent with previous studies showing that extinction of IA memory requires 3 to 6 non-reinforced test sessions with intervals between them ranging from 1 h to 1 d^60,61^. Control groups showed normal decay of IA memory due to the passage of time (Figs. 1, 3 and 5). Retention scores at 1, 7 and 14 days were similar to those observed in experiments where independent groups of animals were tested thrice^21,22,62^.

### Open field task (OF) task

The arena (50 cm wide × 50 cm long × 39 cm high) was made of black plywood walls and floor divided into nine squares by white lines. For the task, each rat was placed in the central zone of the arena and freely explored for 10 min. During this period, time spent in the center and the number of rearings were registered^63^.

### Elevated plus maze (EPM) task

The apparatus was made of acrylic and had two open white arms (50 cm long × 10 cm wide) and two closed black arms (10 cm wide × 50 cm long × 40 cm high). It was 1 m elevated over the floor. For the task, each rat was placed in the central square (10 × 10 cm) and freely explored the maze for 5 min. During this period, time spent in open arms and total entries in the four arms was registered^64^.

### Statistical analysis

For IA experiments, data were analyzed using: Mann–Whitney U test for comparison between control and experimental groups for each test (1 h, 1 d, 7 d and 14 d) separately or Wilcoxon one-tailed signed rank test for comparison between training and test of the same group. For EPM and OF experiments, data were analyzed using Student's t two-tailed test. Significant differences were set at *p* < 0.05. For simplicity, all the results are presented as mean ± SEM. All data were analyzed using GraphPad Prism® version 8.00 (GraphPad Software, La Jolla, CA, USA).

## Data Availability

The datasets that support the findings of this study are available from the corresponding author upon reasonable request.
